# Evaluation of the Cytotoxic and Autophagic Effects of Atorvastatin on MCF-7 Breast Cancer Cells

**DOI:** 10.4274/balkanmedj.2017.0604

**Published:** 2018-05-29

**Authors:** Tuğba Alarcon Martinez, Naciye Dilara Zeybek, Sevda Müftüoğlu

**Affiliations:** 1 Department of Histology and Embryology, Hacettepe University School of Medicine, Ankara, Turkey; 2 Department of Pediatrics, Hacettepe University School of Medicine, Ankara, Turkey

**Keywords:** Apoptosis, atorvastatin, autophagy, breast cancer, cytotoxicity, MCF-7 cells

## Abstract

**Background:**

Recently, cytotoxic effects of statins on breast cancer cells have been reported. However, the mechanism of anti-proliferative effects is currently unknown. Autophagy is non-apoptotic programmed cell death, which is characterized by degradation of cytoplasmic components and as having a role in cancer pathogenesis.

**Aims:**

To investigate the anti-proliferative effects of atorvastatin on MCF-7 human breast adenocarcinoma cells with respect to both autophagy and apoptosis.

**Study Design:**

Cell culture study.

**Methods:**

Cell viability was analyzed using WST-1 cell proliferation assay. Apoptosis was determined by the TUNEL method, whereas autophagy was assessed by Beclin-1 and LC3B immunofluorescence staining. Ultrastructural analysis of cells was performed by electron microscopy.

**Results:**

Atorvastatin reduced MCF-7 cell proliferation in a dose- and time-dependent manner inducing TUNEL-, Beclin-1-, and LC3B-positive cells. Moreover, ultrastructural analysis showed apoptotic, autophagic, and necrotic morphological changes in treatment groups. A statistically significant increase in the apoptotic index was detected with higher concentrations of atorvastatin at 24 h and 48 h (p<0.05).

**Conclusion:**

The anti-proliferative effects of atorvastatin on breast cancer cells is mediated by the induction of both apoptosis and autophagy which shows statins as a potential treatment option for breast cancer.

Breast cancer in women is a common malignancy around the world. It is the second leading cause of mortality among cancer types in developed countries. According to the World Health Organization, 570.000 women died from breast cancer worldwide in 2015 (1). Currently, treatments for breast cancer are hormone therapy, chemotherapy, radiotherapy, and surgery. However, since some patients do not respond to these treatments, several studies have been conducted to find new strategies to treat breast cancer (2). Statins belong to a set of medications that act by decreasing blood cholesterol levels through the specific inhibition of 3-hydroxy-3-methylglutaryl coenzyme A reductase enzyme. Besides these effects on lipid metabolism, statins induce immunomodulatory, anti-inflammatory, and antioxidant activity (3,4). In addition, during the last few years, antineoplastic effects of statins have also been reported (3,5). Atorvastatin is one of most commonly prescribed statins for the prevention of both cardiovascular and cerebrovascular diseases. Moreover, atorvastatin shows anti-proliferative effects on different cancer cells including breast cancer cells. Therefore, atorvastatin has gained increased interest as a potential therapeutic agent for use as an anticancer treatment (6,7). Although the exact mechanism of its anti-proliferative effects is currently unknown, atorvastatin both modifies the cell cycle and induces growth suppression or apoptosis of malignant cells (7,8). In addition, the lipophilic nature of atorvastatin enables it to cross the cell membrane easily and induce these effects. Autophagy is a type of cell death process by which cells recycle their unessential organelles and components under stress conditions (9). It provides new insight into cell death pathways, especially in cancer where it is thought to have a dual role (10,11,12,13). Thus, autophagy may behave both as a tumor suppressor by preventing accumulation of damaged organelles and misfolded proteins or as a cell survival mechanism by promoting the development of tumors (12). Therefore, understanding the role of autophagy and its regulation in cancer cells may be essential to develop new cancer drugs. Although atorvastatin has been reported to have some autophagic effects on different kinds of cancer cell lines, there is no evidence in breast cancer (10,14). Our article on autophagy examined the anti-proliferative effects of atorvastatin on breast cancer MCF-7 cells.

## MATERIALS AND METHODS

### Cell line and cell culture

**Figure 1:**
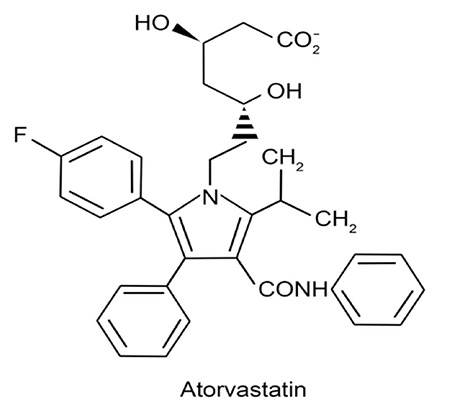
Chemical structure of atorvastatin.

For MCF-7 experiments, a human breast cancer cell line (DSMZ, Germany) was used. RPMI1640 (Sigma Chemical Co., St. Louis, MO, USA) was used for cell culture experiments. The culture media was maintained with 10% fetal calf serum, 1% penicillin-streptomycin, and 1% L-glutamine. Experiments were conducted at 37 °C, 100% humidity, and 5% CO_2_ incubator conditions. MCF-7 cells were incubated with atorvastatin (Godecke/Parke-Davis: Freiburg, Germany) at 5, 10, 20, 40, and 80 μM concentrations for 24 h and 48 h. The chemical structure of atorvastatin is represented in Figure 1 (Courtesy of Schachter M., published in Fundamental and Clinical Pharmacology, published by John Wiley and Sons).

### Cell viability determination

Cell viability and cytotoxic effects caused by atorvastatin were determined by a WST-1 assay. This method depends on the cleavage of the stable tetrazolium salt to soluble formazan. The amount of formazan produced indicates the metabolically active cells. Therefore, quantifying the optical density of formazan can determine the cell viability with regard to both metabolic rate and cellular respiration. Each experiment was replicated three times. The percent viability represents the mean of two measurements of cells with respect to untreated control. MCF-7 cell lines (10×10^3 ^cells/well) were seeded into plates, and after sub-confluency of cells, 100 μL of culture medium (control-without statin) and different concentrations of atorvastatin (5, 10, 20, 40, and 80 μM) were added and incubated for 24 h and 48 h. Subsequently, a WST-1 assay was performed as described in our previous work (15). Just as 10, 20, and 80 μM atorvastatin caused a statistically significant decrease in cell viability at 24 h, and 48 h, further evaluation of apoptotic and autophagic effects on MCF-7 cells were determined with these doses.

### TUNEL analysis of apoptosis

Terminal deoxynucleotidyl transferase-kit conjugated with horseradish peroxidase (11684817001, ROCHE, Mannheim, Germany) was used for detection of apoptosis. MCF-7 cells seeded on chambered slides (Lab-Tec, Sigma, USA) were incubated with 10, 20, 80 μM concentration of atorvastatin for 24 h and 48 h. After the treatment, TUNEL staining was done as previously described (16). A light microscope (LeicaDM6000B, Germany) coupled with a camera (Leica DC490, Germany) was used for light microscopic evaluation of TUNEL-positive cells. TUNEL-positive cells were counted, and the apoptotic index [ratio of apoptotic (TUNEL-positive) cells to the total number of cells] was determined by the total number of 100 cells at 400X magnification.

### Analysis of LC3B, and Beclin-1 immunoreactivity

The immunoreactivity of LC3B and Beclin-1 were detected by immunofluorescence labeling. MCF-7 cells were seeded on coverslips for immunocytochemistry evaluation. The doses of atorvastatin at 10, 20, and 80 μM concentrations were added and incubated for 24 h and 48 h. Then cells were fixed with acetone for 5 min at -20 °C and after washed with phosphate buffered saline (PBS) and kept under ventilator for 10 min for drying. After treating with 2% bovine serum albumin and 10% human serum for 1 h, the cells were labeled with anti-rabbit LC-3B (Cat no: ab63817, Abcam) and anti-rabbit Beclin-1 (Cat no: ab51031, Abcam) at a final dilution of 1:100 for 1 h in a humidified chamber. After removal of primary antibodies and washing with PBS, secondary antibody [Fluorescein isothiocyanate-labeled goat anti-rabbit immunoglobulin G (Chemicon 132F)] was applied for 30 min. After washing with PBS, they were mounted with fluorescence mounting medium. Photographs were taken using a DC490 camera (Leica, Germany) attached to a microscope (Leica DM6000B, Wetzlar, Germany).

### Electron microscopic analysis

For ultrastructural analysis, MCF-7 breast cancer cells were incubated with 10, 20, and 80 μM concentrations of atorvastatin for 24 h and 48 h. Subsequently, both adherent and floating cells were fixed in phosphate buffered 2.5% glutaraldehyde solution, pH 7.4, for 4 h. Then, 1% osmium tetroxide in 0.1 M phosphate buffer solution was used for post-fixation. Cells were washed and placed in 2% agar solution, cooled at room temperature, and sliced into small sections. Then, specimens were dehydrated in an ethanol series and were rinsed in propylene oxide. Afterwards, cells were embedded in Araldite-Epon 812 (Cat no: 13940, EMS, PA, USA) and polymerized at 60 °C for 48 h. Semi-thin sections of 1 μm thickness were obtained with an ultramicrotome (Leica ultracut R, Germany). Methylene blue-azure II was used to stain semi-thin sections. Ultrathin sections with 70 nm thickness were taken using an ultramicrotome and were contrasted with uranyl acetate-lead citrate. An electron microscope (JEOL-JEM 1400, Japan) was used to examine sections, and they were photographed with a charge-coupled device camera (Gatan Inc., CA, USA).

### Statistical analysis

For multiple comparisons of cell viability and TUNEL analysis, we used an analysis of variance followed by Tukey’s test and considered p<0.05 as statistically significant.

## RESULTS

### Atorvastatin induces cell death and decreases proliferation in dose- and time-dependent manner in breast cancer MCF-7 cells

**Figure 2:**
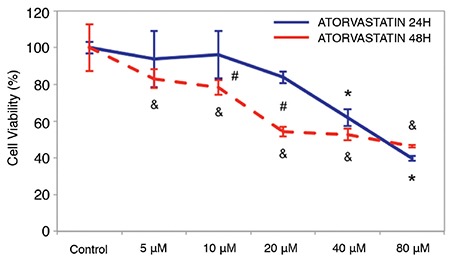
The effect of atorvastatin on cell viability of MCF-7 breast cancer cell line for 24 h and 48 h. Cell viability was assessed by the WST-1 assay. MCF-7 breast cancer cell line was treated with various concentrations of atorvastatin for 24 h and 48 h. Atorvastatin caused a concentration- and time-dependent decrease in cell viability of MCF-7 cell line. Data are expressed as percentage of cell viability with respect to control. Each point represents the mean ± standard deviation of three replicates of two separate experiments. *: statistically different compared with untreated conditions in 24 h treatment group (p<0.05); &: statistically different compared with untreated conditions in 48 h treatment group (p<0.05); #: statistically different compared with 24 h and 48 h

Atorvastatin treatment at 5, 10, 20, 40, and 80 μM concentrations caused a decrease in cell viability of MCF-7 cells after 24 h and 48 h (Figure 2). The difference in the cell viability of untreated and treated MCF-7 cells with 40 and 80 μM of atorvastatin was statistically significant at 24 h (p<0.001, p<0.001) and 5, 10, 20, 40, and 80 μM of atorvastatin were also statistically significant at 48 h (p<0.05, p<0.05, p<0.001, p<0.001, p<0.001, respectively). Further analysis showed that the cell viability of MCF-7 incubated with 10 and 20 μM atorvastatin was statistically significantly lower at 48 h compared with 24 h (p<0.05, p<0.001, respectively; Figure 2).

### Atorvastatin induces apoptosis in MCF-7 breast cancer cells

**Figure 3:**
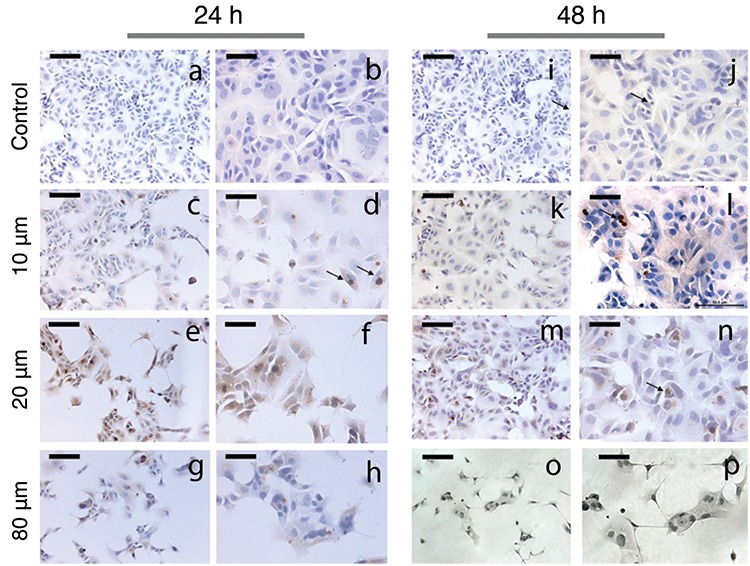
Representative TUNEL-stained sections of MCF-7 cells untreated (a, b, i, j) and treated with 10 μM (c, d, k, l), 20 μM (e, f, m, n), and 80 μM (g, h, o, p) atorvastatin for 24 h and 48 h. Arrows indicate TUNEL-positive cells with brown nucleus (TUNEL assay immunoperoxidase, hematoxylin, Scale bar of a, c, e, g, i, k, m, o: 100 μm; b, d, f, h, j, l, n, p: 50 μm).

**Figure 4:**
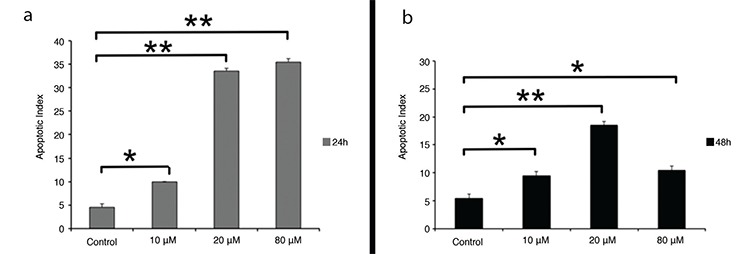
The apoptotic effect of atorvastatin on MCF-7 cells after 24 h (a) and 48 h (b) treatment. Apoptosis was assessed by the TUNEL method. Data are expressed as mean ± standard deviation. *p<0.05, **p<0.001

A TUNEL assay was used for the determination of apoptosis. The number of MCF-7 apoptotic cells increased after treatment with 10, 20, and 80 μM atorvastatin at 24 h and 48 h (Figure 3). Atorvastatin caused a concentration-dependent increase in the apoptotic index at both 24 h and 48 h. There were statistically significant increases of 2-, 6-, and 7-fold in the apoptotic index with 10, 20, and 80 μM concentration of atorvastatin at 24 h, respectively, compared with control (p<0.05, p<0.001, p<0.001, respectively). Atorvastatin caused a concentration-dependent increase in apoptotic index in 48 h only at concentrations of 10 and 20 μM atorvastatin. The number of apoptotic cells decreased with 80 μM concentration of atorvastatin at 48 h as the number of cells in the plate decreased proportionally to increases in the concentration of atorvastatin treatment (Figure 4). The apoptotic index showed a statistically significant increase with 10, 20, and 80 μM atorvastatin at 48 h compared with control (p<0.05, p<0.001, p<0.05, respectively).

### Atorvastatin induces autophagy in MCF-7 cells

**Figure 5:**
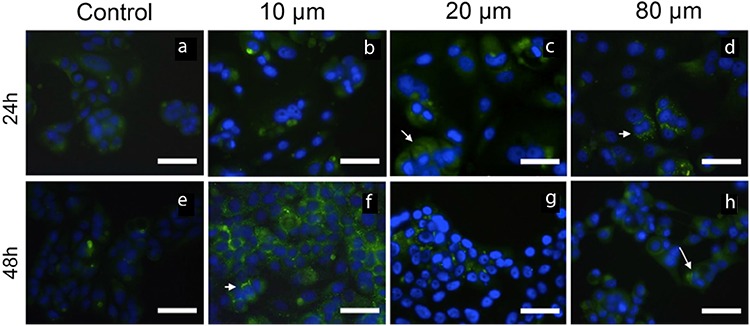
The immunoreactivity of Beclin-1 on MCF-7 cells untreated (a, e) and treated with 10 μM (b, f), 20 μM (c, g), and 80 μM (d, h) atorvastatin for 24 h and 48 h. Arrows indicate Beclin-1-positive cells with green cytoplasmic punctate staining (Immunofluorescence-fluorescein labeled, 4’,6-diamidino-2-phenylindole, Scale bar: 50 μm).

**Figure 6:**
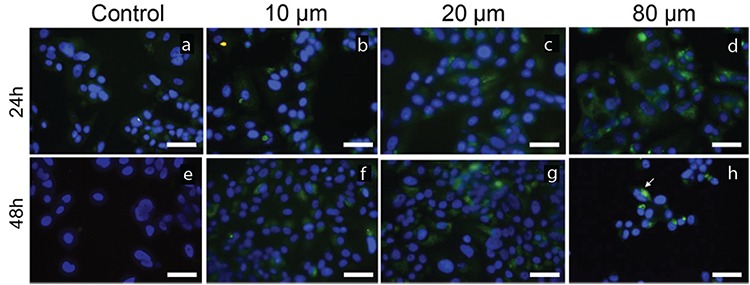
The immunoreactivity of LC3B on MCF-7 cells untreated (a, e) and treated with 10 μM (b, f), 20 μM (c, g), and 80 μM (d, h) atorvastatin for 24 h and 48 h. Arrows indicate LC3B-positive cells with green cytoplasmic punctate staining (Immunofluorescence-fluorescein labeled, 4’,6-diamidino-2-phenylindole, Scale bar: 50 μm).

Autophagy was determined by Beclin-1 and LC3B immunocytochemistry staining. Representative results from immunocytochemistry staining are shown in Figures 5, 6. MCF-7 cells treated with 10, 20, and 80 μM atorvastatin showed immunoreactivity for Beclin-1 and LC3B (Figure 5, 6, respectively). Staining for both LC3B and Beclin-1 was observed as fine granular, punctate forms in intense immunoreactivity. Autophagy was more evident in treatment groups of 10 and 20 μM atorvastatin when compared with that in the control group.

### Electron microscopy results

**Figure 7:**
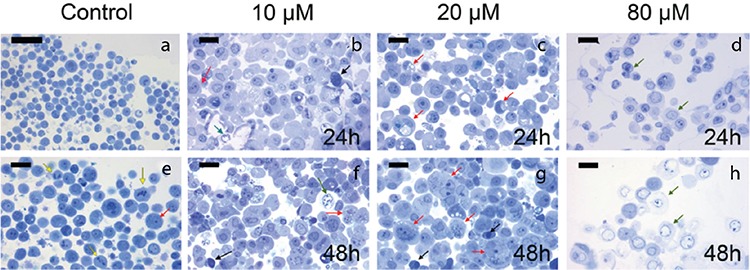
Semi-thin sections of untreated (a, e) and treated with 10 μM (b, f), 20 μM (c, g), and 80 μM (d, h) atorvastatin for 24 h and 48 h. A decrease in the number of cells with normal morphology is seen in treatment groups with increasing concentrations and time intervals of atorvastatin treatment. Yellow arrow: cells in mitosis, red arrow: autophagic cells, green arrow: necrotic cells, black arrow: apoptotic cells (Methylene blue, Scale bar A: 50 μm; b-h: 20 μm).

**Figure 8:**
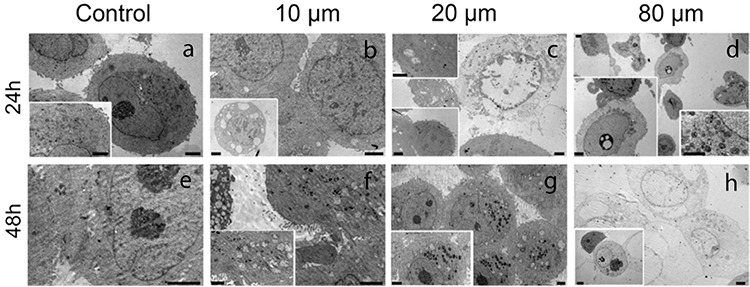
Electron microscopic images of MCF-7 cells untreated (a, e) and treated with 10 μM (b, f), 20 μM (c, g), and 80 μM (d, h) atorvastatin for 24 h and 48 h (Uranyl acetate-lead citrate, Scale bar in left lower inset in c and h: 4 μM, right lower inset in D: 1 μm and remaining are 2 μm).

We observed untreated MCF-7 cells as round cells with a prominent nucleus and nucleolus at different phases of mitosis in semi-thin sections (Figure 7a-7e). A few untreated MCF-7 cells showed vacuoles in their cytoplasm (Figure 7e). MCF-7 cells treated with atorvastatin, especially with 10 and 20 μM atorvastatin for 24 h, had an increased number of cytoplasmic vacuoles in their cytoplasm (Figure 7b, 7c). Some MCF-7 cells treated with 10 and 20 μM atorvastatin for 48 h appeared as giant cells indicating mitotic catastrophe (Figure 7f, 7g). Moreover, MCF-7 cells treated with 10, 20, and 80 μM atorvastatin revealed condensed nuclei of apoptotic cells. Apoptosis was more evident in MCF-7 cells treated with 20 μM atorvastatin for 48 h when compared with cells receiving lower concentrations at shorter time intervals (Figure 7g). An increase in both concentrations and time intervals of atorvastatin treatments resulted in a gradual decrease in the number of cells. After treatment with 80 μM atorvastatin for 24 h and 48 h, most cells lost their normal morphology and showed necrosis (Figure 7d, 7h). Electron microscopy enabled us to detect euchromatic nuclei, prominent nucleoli, vesicles, secondary lysosomes, and numerous rough-endoplasmic reticulum in the cytoplasm of untreated MCF-7 cells (Figure 8). Moreover, indentation and enlargement of the nucleus were prominent resulting in an increased nucleus to cytoplasm ratio (Figure 8a). On the other hand, MCF-7 cells exposed to 10, 20, and 80 μM atorvastatin for 24 h and 48 h showed apoptotic and autophagic degenerative changes (Figure 8b-8d, 8f-8h). After 24 h, MCF-7 cells exposed to 10 and 20 μM atorvastatin showed small vesicles and some vacuolization in their cytoplasm (Figure 8b, 8c). Lipid droplets and vesicles were also increased in MCF-7 cells exposed to 10 and 20 μM atorvastatin for 48 h (Figure 8f, 8g). We observed enlarged pleomorphic vacuoles containing moderate electron-dense material in the cytoplasm of these autophagic cells. However, the presence of both an intact nucleus and nucleolus implied undisturbed cellular integrity (Figure 8g). Some MCF-7 cells exposed to 80 μM atorvastatin for 24 h showed apoptotic changes with apoptotic bodies and nuclear material condensation (Figure 8d-left inset). Prominent cell death was detected in MCF-7 cells treated with 80 μM atorvastatin for 48 h (Figure 8h). Most MCF-7 cells exposed to 80 μM atorvastatin for 48 h completely lost the integrity of their cellular membrane and showed extremely degenerated cytoplasmic content (Figure 8h). Additionally, phagocytic vacuoles with membranous residues and widened perinuclear cisterna were observed in a few apoptotic cells (Figure 8h-inset).

## DISCUSSION

In our study, we examined the mechanism behind the anticancer effects of statins on breast cancer. Recently, beyond lipid-lowering effects, statins were suggested as potential cancer therapeutics (6). *In vitro* studies showed that statins have potent anti-tumor effects in several human cancers including breast cancer (15,17,18,19). Although preclinical evidence demonstrated tumor-suppressive effects, the clinical reports investigating the association between statin usage and breast cancer have yielded mixed results. Therefore, at present, there is a debate about the preventive effects of statins on breast cancer (20,21). Studies conducted with atorvastatin also show contradictory results. For example, in the study by Ji et al. (22) biomarker assessments were not changed by atorvastatin application. However, more randomized clinical trials are needed to investigate these associations. In this study, we showed that atorvastatin displayed anti-tumor activities on breast cancer MCF-7 cells by inhibiting cell proliferation. According to our results, cell viability decreased 60% in MCF-7 cells in a dose- and time-dependent manner in accordance with previous studies, which reported anti-tumor effects for lipophilic statins (7,23). The lipophilicity of statins is an important factor that determines their cellular effects because only lipophilic statins can penetrate the plasma membrane and affect cellular proliferation. Thus, hydrophilic statins do not induce significant anti-proliferative and anti-tumor effects in breast cancer cells (23). For this reason, in our study, we chose atorvastatin, a commonly prescribed lipophilic statin, to evaluate the cytotoxic effects of statins. Furthermore, our results indicated that atorvastatin promoted apoptosis in MCF-7 cells. In this study, various methods like the TUNEL assay and electron microscopic examination for ultrastructural analysis were used for determining apoptosis. It is well known that statins induce apoptosis in various cells lines such as colon, lung, pancreatic, melanoma, prostate, leukemia, neuroblastoma, and breast cancer (5,13,18,19,24). Anti-proliferative and apoptotic effects of statins in breast tumor cells are triggered by inhibiting the enzyme, 3-hydroxy-3-methylglutaryl-coenzyme A reductase. This enzyme is the rate-limiting step in mevalonate synthesis. In addition to the effects on cholesterol biosynthesis, statins regulate the synthesis of various other major products such as dolichol, geranyl pyrophosphate, and farnesyl pyrophosphate. These agents play important roles in cellular functions, including both DNA synthesis and cell cycle progression, and inhibition of their synthesis by statins may induce anti-tumor responses (25,26). However, the anticancer effect of statins acting through a mevalonate-independent pathway is also under investigation. Following atorvastatin treatment, mevalonate and pro-apoptotic pathways are up-regulated in gene expression analyses of breast cancer cell lines (27). To address the biological mechanisms underlying the anticancer effect of statins further, the present study examined subsequent processes of autophagy, apoptosis, and necrosis. In these cells, the activation of autophagy may contribute to apoptosis and/or necrosis in a dose- and time-dependent manner similar to the study in which rottlerin was explored in bladder cancer (11). Autophagy is characterized by lysosomal degradation and recycling of cytoplasmic contents. The cellular homeostasis can be maintained by autophagy with degradation of misfolded proteins and organelles (9). Although autophagy is known as a defensive mechanism in response to cellular stress, the proceeding stimulation of autophagy may also cause cell death, by inducing apoptosis or autophagy (12). Our study demonstrated that atorvastatin-induced autophagy in breast cancer MCF-7 cells. Breast cancer cells showed Beclin-1 and LC3B immunoreactivity and widened perinuclear cisterna, induced by stress depending atorvastatin treatment. Increased vacuolization and engulfment of membrane residues and/or cytoplasm by autophagic vesicles may be attributed to autophagy, and these changes were more prominently observed in MCF-7 cells treated with 10 and 20 μM atorvastatin for 48 h. Our results confirm previous studies that showed autophagic effects of atorvastatin on different kinds of cancer cell lines (10,11,12,13,14). Atorvastatin induced autophagic alterations in MCF-7 cells even at the lowest doses used. In addition, a shift from autophagic changes to both apoptosis and necrosis is detected with gradually increasing atorvastatin concentrations. The results revealed that treatment of atorvastatin induced autophagy and subsequent apoptosis. Importantly, findings of our study highlight an additional mechanism for the anti-proliferative effect of statins on breast cancer cells. Many studies showing autophagy in both tumor and normal cells suggest that statins play a role in the regulation of cancer treatments (13,14,25). Statins are reasonably safe and relatively inexpensive drugs. Once the preventive or treatment effects of statins on breast cancer cells are verified, statins would be available for breast cancer patients as a cost-effective, low-toxicity option for adjuvant therapy in the short term. This study shows atorvastatin as a promising treatment option for breast cancer by activating both autophagy and apoptosis and resulting in the death of tumor cells. In conclusion, several preclinical studies described the anti-proliferative and pro-apoptotic effects of statins on breast cancer cells. Statins are widely considered to have therapeutic potential in breast cancer, although further questions should be answered about their clinical significance. Identification of the precise mechanisms by which statins elicit anti-proliferative effects will directly affect the role of statins in the treatment of breast cancer. The present study revealed that atorvastatin treatment has anti-proliferative, pro-apoptotic, and autophagic effects on breast cancer cells. Statins can lead to autophagy and cell death in breast cancer cells. Further studies should be conducted to understand the relationship between autophagy and apoptosis; however, both the clinical significance and relevance of these findings need to be verified. The inclusion of statins in combination therapies for breast cancer can be a promising new treatment strategy for the future.
